# Moving toward a Handheld “Plasma” Spectrometer for Elemental Analysis, Putting the Power of the Atom (Ion) in the Palm of Your Hand

**DOI:** 10.3390/molecules26164761

**Published:** 2021-08-06

**Authors:** Brian T. Buckley, Rachel Buckley, Cathleen L. Doherty

**Affiliations:** 1 Environmental and Occupational Health Sciences Institute, Rutgers University, Piscataway, NJ 08854, USA; cdoherty@eohsi.rutgers.edu; 2 Department of Chemistry, Indiana University, Bloomington, IN 47405, USA; kpbuckle@iu.edu

**Keywords:** plasma, spectrometer, handheld device design, micro-technology, excitation, ionization, metal analytes

## Abstract

Many of the current innovations in instrument design have been focused on making them smaller, more rugged, and eventually field transportable. The ultimate application is obvious, carrying the instrument to the field for real time sample analysis without the need for a support laboratory. Real time data are priceless when screening either biological or environmental samples, as mitigation strategies can be initiated immediately upon the discovery that contaminant metals are present in a location they were not intended to be. Additionally, smaller “handheld” instruments generally require less sample for analysis, possibly increasing sensitivity, another advantage to instrument miniaturization. While many other instruments can be made smaller just by using available micro-technologies (e.g., eNose), shrinking an ICP-MS or AES to something someone might carry in a backpack or pocket is now closer to reality than in the past, and can be traced to its origins based on a component-by-component evaluation. While the optical and mass spectrometers continue to shrink in size, the ion/excitation source remains a challenge as a tradeoff exists between excitation capabilities and the power requirements for the plasma’s generation. Other supporting elements have only recently become small enough for transport. A systematic review of both where the plasma spectrometer started and the evolution of technologies currently available may provide the roadmap necessary to miniaturize the spectrometer. We identify criteria on a component-by-component basis that need to be addressed in designing a miniaturized device and recognize components (e.g., source) that probably require further optimization. For example, the excitation/ionization source must be energetic enough to take a metal from a solid state to its ionic state. Previously, a plasma required a radio frequency generator or high-power DC source, but excitation can now be accomplished with non-thermal (cold) plasma sources. Sample introduction, for solids, liquids, and gasses, presents challenges for all sources in a field instrument. Next, the interface between source and a mass detector usually requires pressure reduction techniques to get an ion from plasma to the spectrometer. Currently, plasma mass spectrometers are field ready but not necessarily handheld. Optical emission spectrometers are already capable of getting photons to the detector but could eventually be connected to your phone. Inert plasma gas generation is close to field ready if nitrogen generators can be miniaturized. Many of these components are already commercially available or at least have been reported in the literature. Comparisons to other “handheld” elemental analysis devices that employ XRF, LIBS, and electrochemical methods (and their limitations) demonstrate that a “cold” plasma-based spectrometer can be more than competitive. Migrating the cold plasma from an emission only source to a mass spectrometer source, would allow both analyte identification and potentially source apportionment through isotopic fingerprinting, and may be the last major hurdle to overcome. Finally, we offer a possible design to aid in making the cold plasma source more applicable to a field deployment.

## 1. Introduction

There is always a significant need for field transportable instruments, ideally ones that make a reading or measurement in real time with little or no sample prep, and possibly even without highly skilled technicians to operate these devices [1,2,3]. Real-time field measurements provide instant feedback on the situation or event being monitored. Historically, most efforts to expand field transportable instrumentation have been directed toward molecular analysis rather than metal and other inorganic analytes. Real-time biomonitoring screening initiatives that identify populations at risk, or contaminant measurements immediately after a contaminant release, are crucial for assessing the magnitude of damage during environmental catastrophes. Just about any measurement that is made in the laboratory is more utilitarian if it can be made in the field, where response time is often critical. Such examples include measuring blood lead (PbB) levels in children [4] or environmental studies on the dust from the World Trade Center (WTC) on 9/11 [5,6]. These technologies can even be used protectively, e.g., to confirm that a shipment of grain is free of mercury prior to distribution and consumption, or to assess whether a drinking water source contains lead. Real-time measurements at the site of sample collection can save time, money, and potentially avert health risks. While many of the modifications for field transport are targeting molecular analytes, there are other instances (like those above) that could benefit significantly from real-time field measurements of metals. Given all the advances in both field transportable molecular analysis and new atomic sources, high precision metals analysis has recently moved much closer to being field transportable. A successful “handheld” plasma atomic spectrometer may be achievable with current technology for many applications. For the purpose of this discussion, we will refer to instruments that are battery powered and can be easily carried as handheld.

## 2. A History of Plasma Spectroscopic Components

Many of today’s state of the art devices (e.g., dielectric barrier discharge) are actually derived from another plasma device (capacitively coupled plasma). Historically “plasma” emission spectrometry [7] came before all others and was initially driven by arc/spark and flame as the vaporization and excitation sources for metal analytes. Early detectors for emission sources used prisms to separate the metal analyte emission signal, from background emissions associated with the plasma gas(es). Semi-quantitative analysis could be performed with arc/spark sources by capturing the individual emission line intensities on a photographic plate. Subsequently, photomultiplier tubes (PMT) allowed for quantitative analysis of the emission light intensity, and when coupled with a flame atomization/excitation source, true quantitative analysis could be performed for elements that are easily excited by a flame. Eventually, ruled gratings replaced or complimented prisms, and solid-state technologies replaced PMT, making the spectrometers more reliable, if not smaller. Now, spectrometers with spectroscopic resolution rivaling a 3 m monochromator can be made with a footprint slightly bigger than a phone.

Plasma mass spectrometry was created using similar designs to instrumentation targeting organic analytes. Inorganic mass spectrometry predated organic by decades and was responsible for many of the earliest innovations in mass spectrometry [8,9,10]. The ionization sources for inorganic mass spectrometry remain largely unchanged for many years. The coupling of an inductively coupled plasma (ICP) with a mass spectrometer provided the most significant innovation in inorganic mass spectrometry since its inception [8]. Before plasma’s, almost all inorganic mass spectrometric methods had been based on low pressure sample introduction processes. Liquid or gas phase analysis was difficult under these conditions, making the technique almost exclusive to solid phase measurement (e.g., metals and glasses). Today, ICPMS is by far the dominant form of inorganic analysis, demonstrating the utility of developing an atmospheric pressure ionization source for inorganic sample analysis. An ICP ionization source creates the most utility, but also the greatest challenge to mass spectrometric analysis. Mass spectrometric analysis generally requires a very high vacuum, low-pressure environment, both to minimize interferences from non-analytes and to maintain sensitivity by minimizing collisions between ions and neutrals. Multi-stage differential pumping is also required to take an analyte from relatively high gas flow rates, high pressure gradients, and high temperatures associated with the ICP, to the low-pressure conditions of the mass spectrometer. Both liquid chromatographic (LC) and ICPMS interfaces meet these substantial pumping requirements for their respective analyte classes (semi-volatile/non-volatile organic and metals), using specific types and capacities of vacuum pumps.

A high energy ionization source is required to atomize and ionize metal analytes. Photon emission is thought to occur after the atom has been ionized by an energetic plasma species (e.g., Ar metastable) followed by electron-ion recombination. Historically, the commercially available sources have been plasmas, either ICP [11,12,13] or direct current plasma (DCP) [14], and even a microwave induced plasma (MIP) [15,16]. All require substantial power supplies and usually secondary support such as water cooling. None of these things lend themselves to a handheld instrument design. They also generally run off a single gas, so either a gas purification system for nitrogen plasma generation or bottles of purified gas must be used. While a mixed gas or N_2_ plasma is feasible for field transportation/handheld devices, it is the power requirement for plasma generation that presents the most significant limitation.

## 3. Advantages of Miniaturization

To determine whether an atomic plasma emission or mass spectrometric measurement can be made “handheld”, we must examine the limitations to making such an instrument possible. There already exist instruments with many of the desired capabilities, lower power sources capable of atomic fluorescence [17], possibly emission [18], field ready optical and mass spectrometric detectors, and plasma sources capable of using air (before scrubbing) as the plasma gas. Arguably, if some of the other components normally used to build spectrometers (e.g., vacuum pumps, power supplies, gas generators, etc.) could have been manufactured much smaller when these instruments were originally designed, then the early models would have already been much smaller than those we use today. Smaller volumes in many sections of the instrument fundamentally lend themselves to easier, more sensitive measurements as the analyte does not become diluted in large ionization source gas volumes or m/z separation architectures. The only time this argument falls apart is when too many ions in a mass spectrometer create space charge limits, or when non-excited atoms on the outside of the plasma absorb emitted photons from metal analytes in the plasma interior. If all the fundamental processes required to ionize, transport, and separate ions could be carried out at the level of micro-liter (µL) rather than liter (L) volumes, the analyte ions in a sample could avoid needless dilution and routine femtogram (fg) to attogram (ag) measurements could theoretically be possible. While larger volume excitation sources (e.g., ICP) also create a signal dilution problem, optical measurements are easier to work with because the measurements are made at atmospheric pressure.

## 4. Requirements for Excitation Ionization Sources

### 4.1. Potential Ionization Sources

Ion sources for atomic mass spectrometry require the matrix and analyte to be suitably vaporized, atomized, and then ionized. Ions can subsequently be focused into the mass spectrometer to be separated and quantified. Electron ion recombination or relaxation of excited state atoms creates photon emission which can also be quantified. An ICP is a hardy source capable of accomplishing these tasks for all sample types, with varying degrees of efficiency. Conventional ICP sources require significant power, (water) cooling, and generally an inert gas supply. All these requirements make a handheld device essentially unmanageable [19]. Some of these requirements might be overcome or adaptable (e.g., Peltier cooling, gas scrubbers/N_2_ generators, etc.), but even if that is possible, the commercial ICP requires a radio frequency generator operating at high current with a significant gas flow. Consideration of alternative commercial plasma sources also presents challenges. Commercial microwave induced plasmas [20] and surfatron plasmas require very high frequency generators with a significant power supply or relatively large waveguides to launch and sustain the surface wave [21]. If we eliminate the frequency generated plasmas, what remains are the multiple configurations of the breakdown plasmas using DC voltages, preferably using readily available gasses or gas mixtures such as air [22].

#### 4.1.1. Breakdown Plasmas

Breakdown plasmas are created when an optimal electrostatic potential is generated between two electrodes. This induces an electron cascade within the neutral gas molecules, creating a current of electrons between the electrodes. Spectroscopic use of breakdown plasmas was at the origin of the atomic spectroscopy. Variations of the electrode types used to induce the breakdown differentiate one plasma type from another. We have listed several of these breakdown sources below.

#### 4.1.2. Arc/Spark

The earliest form of breakdown plasma, arc, and spark spectrometers were used very early on for atomic emission spectroscopy and were in fact used to generate the emission wavelength tables used in many reference texts such as the CRC Handbook of Chemistry and Physics [23,24,25], and these are also available online (https://physics.nist.gov/PhysRefData/ASD/lines_form.html (accessed on 15 July 2021)). The ion emission wavelengths co-listed with the atomic emission lines demonstrate the capability of these sources to provide ions from analytes, in addition to the excited state atoms. Arc and spark sources can be created with higher voltages and only moderate current, well within the capability of battery power, especially if the spark gap is small. Unfortunately, arc/spark sources suffer a lack of reproducible optical focal point, so the design needs to consider collecting the light in a defocused fashion. Alternatively, a magnetic field could be used to focus the emission [26]. Arc/Spark spectrometers gave rise to direct current plasma instruments for quantifying metals in all types of matrices [27]. A cosine corrected fiber optic inlet could collect the light for use as an emission spectrometer if it is close enough to the excitation source and the spark gap is small. Arc/spark sources are unlikely to serve as an ionization source for mass spectrometry however.

#### 4.1.3. Cold Plasmas, Corona Discharges, and Other Breakdown Plasma

By definition, all plasmas described in this manuscript are breakdown plasmas because a high enough potential is created to force an insulator, in this case an inert gas to breakdown and conduct an electrical current [28]. Other examples of breakdown plasmas/ion sources include glow discharges and the newer non-thermal (cold) plasma sources, primarily used in disinfection for food [29] and wound treatment [30]. Both technologies are limited in their ability to ionize different matrices and are not currently configured to handle solution samples. The glow discharge source also requires a low-pressure environment that does not lend itself to rapid sample changes without significant source modification or replacement of a primary source component. The cold plasma sources currently used for disinfection or wound treatment do not have a sample introduction interface, but lend themselves to the significant modification required to become excitation/ionization sources [31]. They are also close in configuration to the capacitively coupled plasmas (CCP) and the dielectric barrier discharge breakdown (DBD) plasmas [32] described below. They are even now referred to often as DBD [33] and some are operated using DC power supplies and become naturally oscillatory [31].

CCP, DBD and other plasmas described here are what are commonly known as cold plasmas because they are not observed to be in thermal equilibrium. The measurement techniques used to estimate the plasma’s temperature [19] also demonstrate that these cold plasmas are much less energetic than other plasma sources.

The CCP is one of the earliest examples of a breakdown plasma used for analytical chemistry [21]. These CCPs became miniaturized [34] and have a very similar configuration to the dielectric barrier discharges (DBD) [35]. Going forward, CCPs and all other plasmas that do not obey thermodynamic equilibrium will be referred to as “cold” plasmas.

Unlike surfatron plasmas which use dielectrics such as quartz capillaries to launch the plasma generating surface wave, DBDs use electrodes to create the breakdown potential and the dielectrics act as a barrier between the plasma and the electrode. Their application for atomic fluorescence with an eye towards the miniaturization of atomic spectroscopic measurement was reviewed [36]. They are similar in design to the cold plasmas used for wound healing, although these DBDs all use AC waves to generate their oscillatory plasma potentials. Moreover, like those used for wound healing, they not as energetic as ICP, MIP, DCP and others already described. They have primarily been used in atomic emission [37] and atomic fluorescence measurement devices. They do report peak power densities close to the anode, being roughly the same as those of the average ICP [38] but suffer greatly from plasma loading from the solvent. The overall detection limits for both atomic emission [39] and fluorescence [40] are not comparable with ICPMS but perhaps comparable to OES. They have also been used been used even more extensively as sample introduction alternatives for other spectroscopic methods that include ICPMS, AA and ICP OES. An extensive review of the DBD plasma sources and their current applications has been recently published [41].

The DBD plasmas also create a limitation for field transport because they require an oscillatory voltage that would waste power by generating an AC voltage from a DC (battery) power supply. A DBD has been used as an ion source for an ion mobility cell [42,43] but for molecular species only and it was characterized as a soft ionization source. DBDs have not been the primary ion source for any MS device, measuring metals. Many of their atomic emission applications have been adopted in conjunction with hydride generation or other chemical reactants, designed to minimize the matrix effects of most samples. With all of their limitations, cold plasmas have the greatest potential to become a field instrument both because of their lower power draw and their small size [44]. They were first used as atom sources, then for atomic fluorescence.

### 4.2. Voltage over Current: Making the Most of Battery Power

There are many challenges to overcome in creating a field transportable instrument, especially one which can be held in the hand. In a mass spectrometer, the ability to maintain a vacuum (1 Torr to 10^−7^ Torr) is the primary obstacle to overcome while in the ionization source it is the power requirements. In a field transportable instrument with small size, light weight and rugged requirements, the power required becomes a design optimization focal point. Given a choice between high current and high voltage, we would expect that the voltage is easier to create. Any device that is truly handheld and not just a portable appendage tethered to larger non-transportable devices, e.g., power supplies or vacuum pumps, would need to run off batteries. Even with lithium ion or perhaps the next generation batteries, they may not be able to supply enough power for metal analyte ionization, and therefore the excitation/ionization source will need to be a major design element.

### 4.3. Plasma Gases

Commercial plasma devices utilized inert noble gases, primarily argon but also helium. Transporting inert noble gasses to the field is problematic for a handheld device. The ark/spark spectrometers ran in air and accounted for, or subtracted, background emission lines created by nitrogen and oxygen. Minor components generally did not make up enough of a background to require removal. ICPMS spectra usually include molecular ions caused by air gasses or their high energy analyte collision products (e.g., BaO and ArN). These species must be subtracted as potential background interferences. While transporting noble gases with your instrument is impractical, it may be possible to eliminate (or at least greatly reduce) the oxides by nitrogen generation using a scaled down version of the scrubbing systems, generally used for LC/MS applications. While hydrogen has also been used to generate plasmas, electrolytic cracking of water may be possible, but a very unlikely solution. Many of the metal analytes would also form hydrides of the metals which may suppress the formation of the metal cation, crucial in plasma mass spectrometry.

Currently, the commercial microwave plasma uses an ICP like torch but runs off of nitrogen gas, created by nitrogen generators [45]. Nitrogen generators used air as the source gas but scrub it with filters to remove oxygen, water vapor, CO_2_, and other major components of air. Using N_2_ as the plasma gas, created by scrubbing air, lends itself to a field transportable instrument, especially if the plasma source becomes much smaller and the overall gas requirements follow.

## 5. Sample Introduction

Classic ark/spark spectrometry used electrodes (often graphite) to create the spark gap and samples were generally solids, and often powders, that were incorporated into the electrodes or held by them. Later, direct insertion probes were created that allowed a plasma to sample the surface of the probe directly [46] or they used a thermal process to evolve the sample into the plasma for excitation/ionization [47,48,49,50].

Solution samples were nebulized directly into the plasma or directly adjacent to it, allowing the energized plasma constituents to vaporize and excite/ionize the analytes. Occasionally powders were introduced with a gas flow stream using the same pathway as nebulized samples. Gases may present the least challenging state since they can be bled directly into the plasma gas or directed toward plasma. In commercial plasma spectrometers, the samples have generally been reduced to an aqueous solution where it is nebulized and aerosolized, atomized, and finally excited/ionized by the plasma. We can assume a handheld device will be operated in the field where sample preparation is difficult at best.

Sample introduction needs to be highly reproducible if quantitative analysis is to be achieved. Arc/spark spectrometers were generally used for qualitative/semi-quantitative analysis only. The direct current plasma utilized a two-electrode, then three-electrode configuration, with nebulized samples introduced below the confluence point of the plasma, creating a reproducible sample introduction process that allowed for the quantitation of aqueous/liquid samples. Inductively coupled plasmas (ICP) created an annular plasma by punching a hole through the center of the plasma with a nebulized sample. Microwave plasmas were originally commercialized as detectors for GC effluent, quantifying vapors and gases only, but they are currently used for OES [51] and are also energetic enough to ionize metals for MS measurement [52]. Other applications and sample types exist for all these plasma sources but have largely remained in the research area only.

If a handheld spectrometer is to be constructed, a novel design for sample introduction will need to be created. Flow rates of carrier gas and aqueous sample solutions will naturally be reduced to prevent saturating and extinguishing the plasma during the desolvation and atomization processes. Using probes for sample introduction may allow for introduction of either solid or liquid samples, and perhaps even pre-concentration of a metal analyte from a solution matrix, just before analysis. Alternatively, many of the classic solution introduction devices (e.g., nebulizers and spray chambers) are scalable. As long as the plasma is the excitation/ionization source, all manner of solution and solid sample types may be possible. Currently, cold plasma sources are not designed to accept sample solids although capillary plasmas, similar to cold plasmas, have been used for the excitation of organic molecules [3].

**Figure 1:**
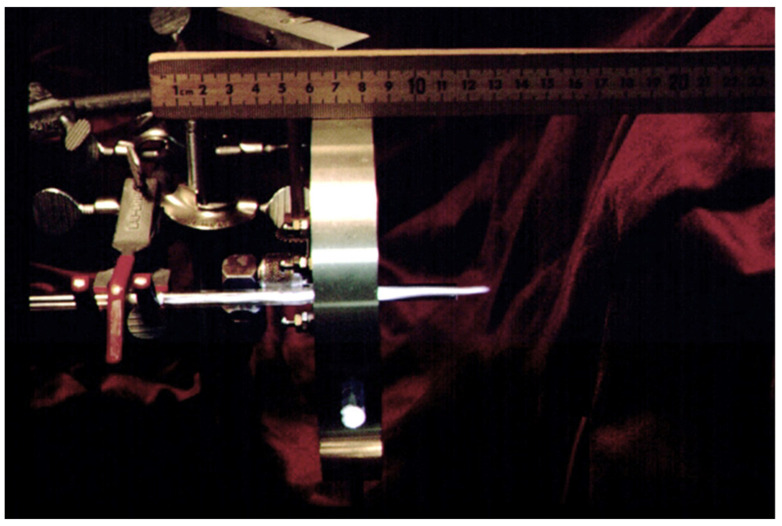
Ar plasma ignited in TM010 Beeknakker microwave cavity powered with 2.54 GHz generator displaying the plasma plume operating well beyond the end of the quartz capillary, possibly used for solid sampling.

Converting arc source to the direct current plasma (DCP) [53] required controlled gas flows around the energized electrode as they were originally used as vapor detectors [54]. If the plasma is energetic enough, nebulized sampled could be introduced into this gas flow and then excited by the discharge. If the analyte is airborne, gas could be sampled that contains either the vapor or particles [55], which include the metal analyte. While there has been work done on ambient sampling for mass spectrometry [56], the methods use a “softer” source (e.g., DESI) and have been primarily directed at molecular mass spectrometry [57]. There has even been a handheld version [58]. The technique does however suggest an answer for solid sampling, specifically to allow the plasma to sample the solid from a probe or directly from the bulk sample, without preparation. Plasmas that extend beyond their containment quartz-ware (torch) have the potential to become a solid sampling device in much the same way a DESI system samples solids. MIP sources have this capability (see Figure 1) and some of the “cold” plasma devices also have that capability as they were originally designed to allow the plasma to contact the surface of the skin or food [59]. However, even the spectroscopic breakdown devices have not been used for solid sampling at the time of this article.

Laser ablation may also be possible from a surface, provided a powerful enough laser diode operated under ambient conditions and within the power capabilities of the power supply. Such devises have been used for outer space-based MS instruments [60]. Recently (patent 2016), a handheld laser induced breakdown spectrometer (LIBS) used a diode pumped solid state (DPSS) LASER as both the sample ablation and excitation source, and this is the closest we have to the theoretical instrument described, but it is presently limited to solid samples and is a dedicated application instrument [61].

## 6. Detectors

Field transportable detectors for plasma sources both optical and mass spectrometric (MS) have existed for decades [62]. Currently, they are not the technology limit for creating the handheld plasma spectrometer as commercial versions for both are readily available, although the commercial optical detectors are generally much smaller than the MS detectors and are significantly less expensive. For the remainder of this manuscript, ion/emission and mass/optical spectrometers will be the principal focus of the handheld devices under consideration. The current technology limit as described above for a handheld plasma MS instrument remains, namely the ability to generate ions efficiently for metal analysis with cold plasmas [44].

### 6.1. Optical Spectroscopy

Spectrometers are required in any type of emission-based atomic measurement, to separate the emitted light signal from background and differentiate one metal’s signal from another [63]. Miniaturization of emission/optical spectrometers grew out of the revolution in solid state camera technologies, providing optical quantitation without sacrificing wavelength resolution for size. While most of the handheld devices were originally focused on IR and NIR measurements that include Fourier-transform (FT) spectrometers [64], a more recent focus has been on visible light [65,66]. The availability of fiber optics and computer generated 3-D printed components has allowed for homemade low-cost spectrometers from simple CCD cameras [67,68,69]. Devices such as those marketed by Ocean Insight have supported the creation of a handheld optical spectroscopic instrument out of any light source that carries data and have already been employed for plasma spectrometers [70]. They have sub-nanometer resolution [71] and today you can purchase a spectrometer for your phone [72]. There are cell phone spectrometers that use sunlight for the source with a cuvette to measure absorbance [73]. These cell phone-based spectrometers are being used for clinical studies as well [74]. As long as collection of the portion of the emission signal that describes the analyte is achieved, the size or capability of the spectrometer should not be the limiting factor. They have even created a device using a micropipette as the scaffolding for the sensor [75]. However, optical-based spectrometers are not as sensitive as mass spectral measurements.

### 6.2. Mass Spectrometry

Depending on the ionization source, mass spectrometric measurements can generally handle solids, liquids, and gasses, do not suffer from the same interferences as these other methods, and are more sensitive. They have other advantages such as isotopic ratio measurements and ion counting, which increases sensitivity. Most MS platforms being miniaturized (e.g., ToF or ion trap) have either high resolution or MS^n^ capabilities. Either of these lessen the effects of interferences and create multi-element capabilities within a single assay. Isobaric interferences still create a problem that remain beyond the capabilities of most handheld instruments, at least for now.

There have been multiple applications [1] and reviews of mini mass spectrometers [76], with most potential candidates for the theoretical instrument proposed here. Specifically, a review of handheld devices by [77,78,79] included field ready instruments and [80] those designed for outer space.

There are several approaches taken by mass spectrometrists to measure the mass of an analyte ion. The first of these techniques, that was useful in advancement towards measuring mass in a handheld manner, is the ion trap [81]. This device is detailed in other works [82,83], however a brief explanation follows. Ions are introduced to oscillatory electric fields. These fields are generated from a symmetric geometry of metallic electrodes. A select range of mass to charge ratios will become stable when the frequency, and magnitude of the voltage, of the oscillations are just right. This “band of stable ions” can be manipulated to ensure that only one specific type of ion is able to transmit through a device. Similarly, if electrostatic voltages are applied to the end of this geometry, ions can be trapped for long periods of time within the stable region, allowing for different mass analysis experiments to be conducted [83]. Another advantage of this device is its relative stability. From an engineering perspective, the ion trap is a more rugged device that can be designed with portability in mind, ensuring stable measurements in the field. They have even been operated at pressures approaching 1 Torr [84].

Ion traps have a distinct set of advantages and disadvantages. The first advantage they have is their ability to operate at relatively “high” pressures for ion detection devices. Similarly, since a stable oscillatory field is all that is necessary, ion traps have been constructed on smaller scales. Several field transportable ion trap devices already exist [85]. A significant disadvantage to miniaturization is the performance of the trap. Performance in this case is defined as mass resolution, or the ability to separate two distinct but similar masses. The performance is directly related to pressure stability as well as longer lengths of filtration, both of which a field transportable device sacrifices for portability.

Another mass analysis approach employed for field instruments, is time-of-flight (ToF) mass analysis. This technique relies on very basic physics relationships to relate the energy of an ion, as well as the time it takes to traverse a known distance, to determine its mass and charge. An advantage of this device is its higher resolutions than ion traps. A significant drawback of these devices towards field portability, is their reliance on higher vacuums. Pressures need to be in the 10^−7^ Torr ranges, which are often difficult to achieve without large diffusion or turbo pumps. Similarly, in a field transportable lens, the electrode configurations are incredibly fragile and must be properly distanced and maintained for accurate measurements.

Another alternative is to relate the collision cross sectional area of an ion of an analyte to its mass to charge. Utilizing a technique known as ion mobility spectrometry (IMS), ions can be separated by means of electrostatic fields. At medium vacuums, the velocity of ion transport is dictated by a variety of factors. Importantly, some of the more critical are the magnitude of the electric field, the charge of the ion, the mass of the ion, and the collisional cross section area of the ion. IMS devices are used extensively for the commercial testing of specific analytes [86]. However, their mass accuracy for unknown analysis is poor. A significant advantage is that this technique works well in parallel with other mass analysis techniques [87]. Moreover, the technique can be performed at atmospheric pressures and already has cheap and rugged designs already created [88,89].

Utilizing this explanation, the best approach would likely be to construct an IMS-ion trap device. The advantages towards field portability of this device have recently been reviewed [90] but include atmospheric pressures that could be utilized to conduct IMS separation. The high voltages necessary to perform the separations can be generated easily without drawing massive power loads. The IMS device can be rugged and small (on the order of approximately 3 inches) to perform a crude separation of the array of ions generated from the plasma. Ions would be electrostatically gated to isolate one specific “time” that ions would take to traverse the IMS separation region. These ions would be selectively pulsed into the ion trap device where their mass to charge ratio would be measured. The advantage of this configuration is the perpendicular analysis of both drift time as well as mass to charge, which has been shown to dramatically increase peak capacity in normal analysis [91]. Similarly, if the spaces are generated correctly, a small roughing pump would be all that is necessary to remove enough gas from the ion trap to perform the mass measurement, while the drift separation could be performed at atmosphere, requiring no pumping. A review that includes many of the commercial instruments discusses the ion trap on a chip approach among others and many of its limitations, but all of the instruments were created for molecular analytes [92] or an ion trap-based palm spectrometer [93]. Although primarily used for vapor or gas detection, multiple vendors now report a commercial, transportable, battery powered IMS or mass spectrometer (https://www.bayspec.com/spectroscopy/portable-mass-spectrometer/; https://api.inficon.com/v1/attachment/b0ddf534-db3e-4920-b9c1-ec872bc28a4d (accessed on 15 July 2021)). Laboratory constructed instruments have also been reported [85] as well as ones used in the field [94,95], but not yet for metals. It would appear that a mass spectrometer for use as a detector is within reach, as such devices continue to become smaller [96] and require much lower voltage [97].

If measurement of the analyte signal, either emission or ion, is already capable with handheld devices or within reach, then sample introduction and analyte excitation/ionization are the primary challenges in creating a handheld instrument and the focus of further discussion.

### 6.3. Need to Incorporate Appropriate Supporting Technologies

There are many additional support requirements to running an ICPMS (e.g., cooling gases, inert plasma gases, etc.). Field transportable mass spectrometers have generally focused on being more rugged and made for installation into a field transportable laboratory [98], rather than a true field ready device. High power devices, and those that require significant (two-stage) vacuum pumping like an ICPMS, by default cannot be considered handheld. Additionally, those that require additional support such as water cooling or inert plasma gases present lesser challenges, but still need to be considered in any design. The limitations to creating a handheld ICP-MS appear to be insurmountable. Given the limitations, it is more likely that a handheld plasma mass spectrometer will not utilize an ICP ion source and perhaps not a conventional mass spectrometer either, although the current advancements in the miniaturization of mass spectrometers may allow for a handheld version in the very near future.

## 7. Competing Technologies

There are already non-plasma-based competing technologies in the field that are capable of quantifying metals and making other physiological and clinical measurements directly and in real-time. Some of which include electrochemical methods such as the anodic stripping voltammetry (ASV) [99] routinely used for blood lead (PbB) measurement, x-ray fluorimeters (XRF) and laser induced breakdown spectrometers (LIBS) for measuring higher concentration metals in solid samples, ion selective electrodes for various metals in solution, and colorimetric methods using premade reagent kits [100].

LIBS uses the energetic photons from a laser to create a plasma on the surface of the sample, melting, vaporizing, and exciting the analytes, arguably acting as a cold plasma spectrometer. It has been made field transportable and applied to solid matrices [101] and also has handheld versions [102]. LIBS requires no real sample preparation but unfortunately does not do liquid samples without additional devices [103] (and it suffers from non-reproducible shot-to-shot matrix breakdown, making quantification much more difficult. True matrix matching is essential as is compensation for variation in signal using additional excited state species for plasma normalization [61]. Field transportable LIBS was recently reviewed [104].

A principal competitor with LIBS for handheld elemental analysis is XRF. XRF uses x-rays to excite the analyte metal and then quantifies the photons emitted once the analyte metal relaxes to a less excited state. Like LIBS, XRF, especially the “handheld” versions are designed for screening metals/alloys in environmental and geological samples (i.e., solids), but have detection limits roughly in the ppm to parts of percent’s range [104]. While x-ray fluorescence can excite many elemental analytes, they are often used for single element analysis of solid-state samples (e.g., Pb in household paint). One limitation is the significant drop in fluorescence yield with decreasing atomic number and the corresponding drop in sensitivity for the lighter elements. There are also licensing requirements, special operating precautions, and possible transportation restrictions as well as operator safety training because of its open beam X-ray. Finally, they suffer from the same limitation of a need for a true matrix matched standard material since all readings are taken in-situ.

All of these techniques have very different specificities, sensitivities, and interferences, some much worse after they were made field ready. For example, ASV routinely has detection limits at the ppb level in the laboratory [105] even sub ppb for lead [106], but the field instrument for blood lead measurement reports 1.4 ug/dL. XRF is about 1ppm in a solid sample in the lab but a single to hundreds of ppm in the field [107] and 15–20 ppm in water for copper and lead [108].

Electrochemical methods that employ ion selective electrodes (ISE) now have multiplex capabilities and solution detection limits in the ppb range [109] but require analytes in solution or conductive matrices and suffer from significant concomitant ion effects [110]. Their selectivity is determined by a difference in permeability in the electrode membrane, passing the analyte ion in favor of concomitant ions. Another example, the electrochemical detectors for PbB have also been used in the field but were subject to significant analytical bias The LeadCare system that employs ASV has been recommended not to be used in research studies [111] because of its lack of sensitivity and perhaps negative bias. Eventually, this device will be recalled [112]. All existing commercial technologies suffer from one limitation or another when compared to a plasma spectrometer.

Comparing sensitivities between instrumentation or platforms is never easy, detection limits are driven by matrix sample preparation (e.g., preconcentration) sample introduction (e.g., chromatographic analyte isolation) laboratory based vs. field transportable and available information. Many authors do not include their detection limits as pertinent information when describing their application. Generally, ICPMS and ICPOES sensitivities are the most sensitive for solution samples with ICPMS now exploring single particle or single cell measurements [113,114]. However, they are not field transportable. With special preparation schemes, XRF and LIBS have been approaching ICPOES sensitivities with field instruments [115,116]. ASV has also been reporting sensitivities to rival ICP OES with laboratory instrumentation [117].

Solid sample analysis is more competitive among techniques as the total dissolved solids for a nebulized solution throttle the detection limits for the plasma-based technologies and most instrumentation field and laboratory seem to have detection limits in the ppm range. Both field transportable XRF and LIBS instruments were originally designed for assessment of impurities in steel with their application to other matrices a natural progression of necessity mothering invention. With these constraints in mind, Table 1 is presented as a very rough survey of detection limits for the various techniques discussed, so that relative sensitivities could be compared. It is a mixture of both older and very recent references and for approximate comparisons only. No real distinction was made between the terms limit of detection (LOD) or detection limit (DL) and some had to be converted from molar concentrations to weight/volume DLs and all values are ppb, either ng/mL or ng/g. What is apparent for a literature search is that not all reported values have been updated since their original work (e.g., ICPOES) because applications drive most of the work rather than fundamental operation studies. The table contains values reported for multiple applications and both field ready and laboratory instruments grouped generally by solid, water, and blood (special application for Pb). While ICPMS and OES require sample dissolution for analysis, extensive sample preparation techniques were rarely included, with notable exceptions being Cr for oxidation state speciation and some of the water analysis preparations for XRF. Extraordinary sample preparation, such as solids for ASV or ISE, was excluded. Of note by its absence is ISE for blood Pb measurement, as both sensitivity and matrix interferences have kept this application out of the literature.

## 8. Putting the Pieces Together on a Plasma Instrument Feasibility

Not long ago, the power supply for ion sources and axillary support services (i.e., water cooling, inert gas, etc.) made the possibility of a handheld device seem like an insurmountable task, but commercial technologies currently exist, even though they have limited capabilities. For example, the ability to ionize in cold plasmas has yet to be demonstrated with high enough efficiency across the entire mass range and they also suffer from more molecular interferences than their hotter plasma cousins. If, however, commercially available spectrometers/detectors (both optical emission and mass spectrometric) can convert the signal generated by a cold plasma source into a metal analyte concentration via field calibration, then all that is missing is a more energetic or efficient excitation/ionization source. A field transportable ionization source must then be created for this instrument to function as intended, with capabilities for gaseous, liquid, and solid sample analysis, while being powered by batteries. To meet this requirement, it should (1) not require water cooling of the plasma or its power supply, (2) have a moderately low current draw to minimize battery depletion, (3) operate with a fixed rather than oscillatory potential, (4) be able to run on air or possibly nitrogen with inline scrubbers, (5) be energetic enough to ionize most metals of interest, and (6) have a sufficiently stable plasma that is not overwhelmed by the direct injection of aqueous solutions and ablated solids.

The earliest plasmas, arcs and sparks have potential as they can be created with relatively low power and can be generated in air. With the right preparation, they are capable of handling solid-state samples, but they did not have any way to easily accept nebulized or free flowing aqueous samples before the introduction of the direct current plasma arc. Alternatively, surface wave launched plasmas were recognized as early as 1959 [151] and present another possible plasma configuration. A more “modern” configuration was described by Moisan [152] and eventually patented in 1975 [153], later known as the surfatron [154,155]. Surfatron plasma configurations are annular because they are launched along a dielectric material, typically a quartz capillary tube. This configuration lends itself to both low flowrate solution sampling and analyte excitation but requires a microwave or other high frequency power supply (27 MHz–10 GHz) to launch the surface wave [156]. While microwave plasmas have previously demonstrated an ability to handle nebulized samples [157], their power supplies do not currently exist in low power configurations, necessitating consideration of other plasma sources. A MIP has recently been reported that meets all of these requirements and whose power supply is reported to be operated with a 28 V battery [158]. This device does use argon for the plasma gas and a vapor generator for sample introduction, both of which would severely limit its applicability as a field instrument.

Cold plasma devices have been employed everywhere from wound healing [159] cancer treatment [160] to disinfection of food [161] and surfaces [162] or air [163]. Spectroscopic applications used oscillatory power supplies which are much less efficient for battery operation. They also lack the sustained power for efficient ionization. The cold plasmas used for disinfection can be operated using lower DC powers and are capable of sustaining their own self-oscillatory regime [31]. One of the limitations in the current cold plasma excitation sources is the power lost to desolvation and atomization processes. With the right configuration, perhaps a nebulizer could be converted to a cold-plasma pulsed excitation source which draws milliamps of current, but for only tens of µsec/ pulse when operated at approximately 5 kV. A similar device [31] produced rotational temperatures of greater than 2800 K and n_e_ of approximately 10^11^/cm^−3^. While these temperatures and densities are considerably less energetic than a commercial ICP source, it is possible they can be increased with an amplification of the operating voltage. The authors observed a direct relationship between n_e_ and applied voltage [31]. Rotational temperatures are measured using the optical emission of key molecular species and assume a Boltzman distribution of energies [164]. They were also not far from some of the rotational temperatures observed in microwave plasmas [165] and may in fact be energetic enough to excite some analyte metals and produce ions [157]. While this device was operated with He as the plasma source gas, most cold plasma devices can operate with either Ar [166] or air [167] or nitrogen [168].

**Figure 2:**
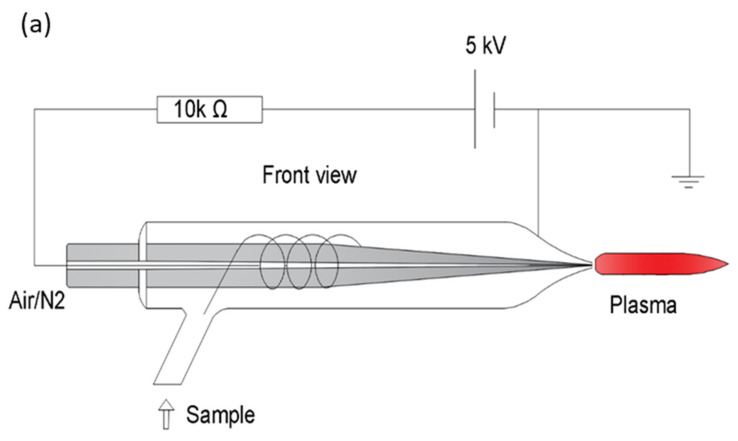

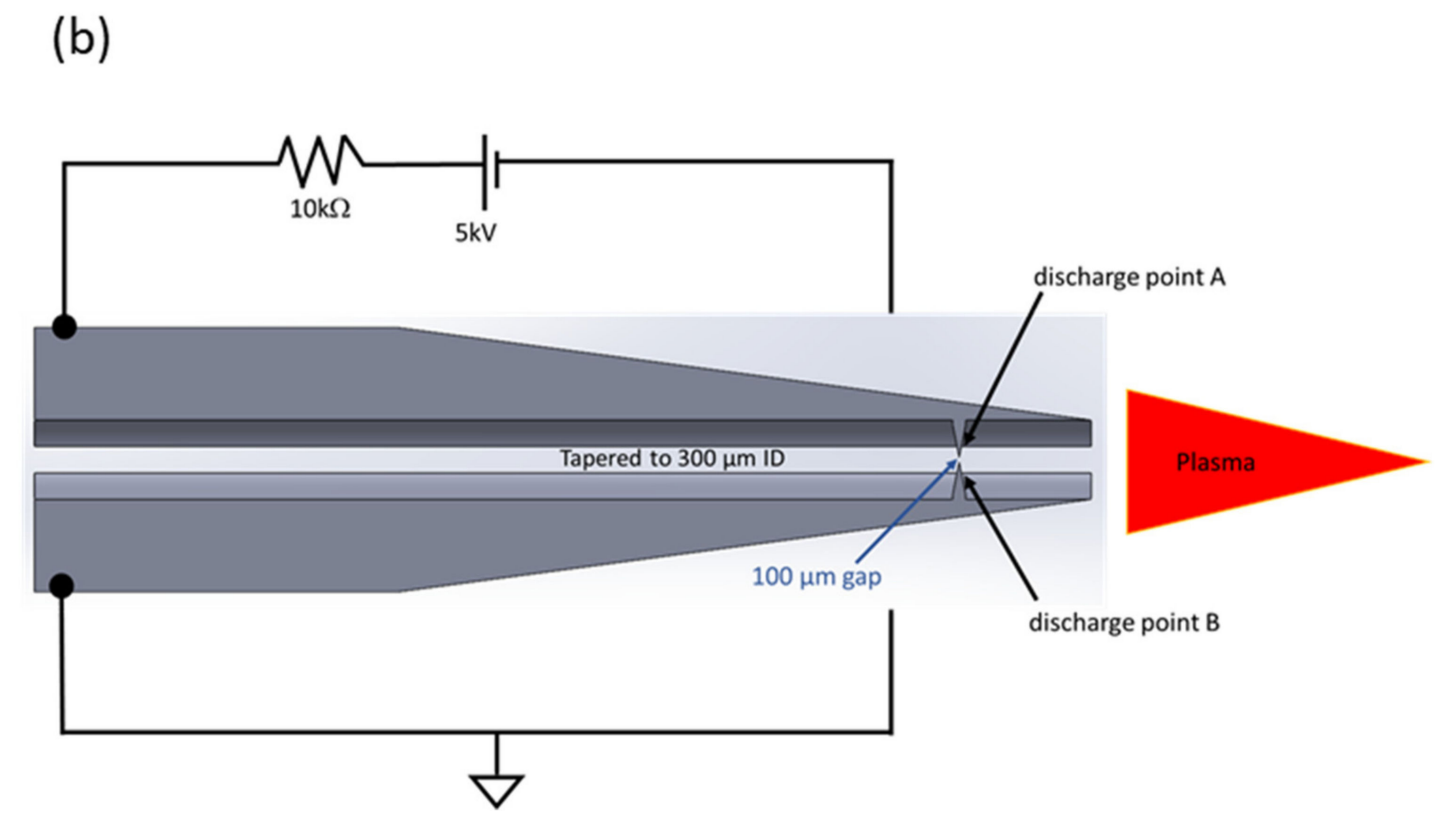
(**a**) Schematic diagram of possible cold plasma source incorporating a corona discharge needle (shown in 2b) as an insert with a side-arm for nebulized sample introduction as part of the design. The overall device would be <10 cm total length. (**b**) A schematic diagram of a corona discharge needle as the potential cold plasma source to be used either as an insert into a larger system or directly with very low sample flow rates. A discharge is generated between the two corona needles on each side of the tip. The discharge can then be used to generate a plasma from the concentric flow gas flow present surrounding the tip.

The great big if: If a cold plasma jet device (perhaps a corona discharge along the sample introduction tube) can be maintained using a concentric flow apparatus, like most capillaries used for ICP spectroscopy (Figure 2), and if the solution is introduced either orthogonally (side-arm Figure 2a) as described by Motley and Long [169], flowing concentrically around the anode it may have enough time to be desolvated and atomized before reaching the hottest part of the plasma plume. Alternatively, if the nebulized solution flows through the central channel of the corona discharge needle (Figure 2b) it may be able to handle µL/min flow rates of solution and be ionized, as earlier direct injection nebulizers have in the past [170]. Solid sampling may be achieved by allowing the plasma jet to come in contact with the sample similar to a DESI source [171] or the direct insertion work for solids in plasmas by arcs [172] and graphite cups [173]. This sampling is possible because most of the cold plasma jet devices produce a plasma that extends beyond its excitation electrodes. If the right configuration of plasma gas flow, applied voltage, torch geometries (e.g., length of concentric tube), and dielectric material can be optimized, a handheld plasma spectrometer capable of analyte ionization may be possible.

## 9. Conclusions

A handheld atomic spectroscopic instrument has many potential applications and will be able to justify any future developmental efforts currently under way. Other techniques approximate handheld methods, but they suffer from multiple interferences, sensitivity deficiencies, and are limited by the type of matrix that can be analyzed. The rapid movement to create mini mass spectrometers, and the success of existing handheld optical spectrometers, suggest that signal measurement is within our reach if a suitable ionization source can be created. Naturally, power limitations for handheld devices preclude the use of a commercial ICP. Alternatively, earlier models of ion/emission, ark/spark, or even the more recent breakdown sources “cold” plasma technologies are already being used for optical emission sources and may provide the blueprint for an ionization source for a handheld atomic spectrometer. Most if not all the technologies currently exist, but the initiative needs to be taken to overcome the remaining hurdle, namely an excitation/ionization source energetic enough for elemental analysis, if a handheld device is to become as utilitarian as the laboratory counterparts.

## Figures and Tables

**Table 1:** Comparison of detection limits reported as ppb (ng/mL or ng/g) for multiple instruments in either solid, water, or blood matrices.

		Analytical Method					
Element	Matrix	ICPMS	ICPOES	LIBS	XRF	ISE	ASV/LeadCare
Pb	Water	1.5 [118]; 0.0042 [119]	2 [120]	0.32 * [116]	0.9 [115]	0.012 [121] (buffer)	0.05 [122]
Pb	Blood	0.06 [123]	1.5 [124]	N/A	N/A	10,000 ** [125]	0.2 [106]; 14 [4]
Pb	Solid	100 [126]	4400 [127]	42,000 [104]; 125,000 [128]	5 [129]; 5000 [130]	N/A	10 [131]
Cr	Water	3 [132]	0.2 [133]	700 [133]	2 [134]; 0.6 [135]	0.4 [109]	0.1 [117]
Cr	Solid	4 (Cr III); 4.5 (Cr VI) [136]	5000 (Cr VI) [137]	17,000 [138]	2000 [139]	N/A	N/A
Cd	Solid	1.8 [140]	500 [141]	4000 [142]	46,000 [129]	N/A	N/A
Cd	Water	1.2 [118]	0.14 [115]	500,000 [143]	4.9 [115]; 0.04 [144]	11 [145]	0.03 [122]
U	Water	0.001 [146]	0.69 [147]	19,000 [148]	17 [149]	13 [150]	0.3 [117]
U	Solid	480 [146]	6000 [141]	250,000 [104]	10,000 [149]	N/A	N/A

* External device (i.e., wood slice) was used as part of the sample preparation. ** DL not reported. Value represents the lowest spike recovery. N/A = values not available in the literature.

## Data Availability

All data (exception concept figure and device description) was obtained from the listed references.

## References

[1] Zhang J., Rector J., Lin J.Q., Young J.H., Sans M., Katta N., Giese N., Yu W., Nagi C., Suliburk J. (2017). Nondestructive tissue analysis for ex vivo and in vivo cancer diagnosis using a handheld mass spectrometry system. Sci. Transl. Med..

[2] Ewing R.G., Miller C.J. (2001). Detection of volatile vapors emitted from explosives with a handheld ion mobility spectrometer. Field Anal. Chem. Technol..

[3] Wolf J.-C., Etter R., Schaer M., Siegenthaler P., Zenobi R. (2016). Direct and sensitive detection of CWA simulants by active capillary plasma ionization coupled to a handheld ion trap mass spectrometer. J. Am. Soc. Mass Spectrom..

[4] Ettinger A.S., Leonard M.L., Mason J. (2019). CDC’s Lead Poisoning Prevention Program: A long-standing responsibility and commitment to protect children from lead exposure. J. Public Health Manag. Pract. Jphmp.

[5] Kostrubiak M. (2018). World Trade Center Dust: Composition and Spatial-Temporal Considerations for Health. World Trade Center Pulmonary Diseases and Multi-Organ System Manifestations.

[6] Durmus N., Pehlivan S., Zhang Y., Shao Y., Arslan A.A., Corona R., Henderson I., Sterman D.H., Reibman J. (2021). Lung Cancer Characteristics in the World Trade Center Environmental Health Center. Int. J. Environ. Res. Public Health.

[7] Sobolev N., Boumans P.W.J.M. (1966). Theory of Spectrochemical Excitation.

[8] Gray A.L. (1974). It all depends on the source. Proc. Soc. Anal. Chem..

[9] Date A.R., Gray A.L. (1981). Plasma source mass spectrometry using an inductively coupled plasma and a high resolution quadrupole mass filter. Analyst.

[10] Houk R.S., Fassel V.A., Flesch G.D., Svec H.J., Gray A.L., Taylor C.E. (1980). Inductively coupled argon plasma as an ion source for mass spectrometric determination of trace elements. Anal. Chem..

[11] Reed T.B. (1961). Growth of refractory crystals using the induction plasma torch. J. Appl. Phys..

[12] Webb B.D., Denton M.B. (1986). Comparison of a very high frequency 148 MHz inductively coupled plasma to a 27 MHz ICP. Spectrochim. Acta Part B At. Spectrosc..

[13] Fassel V.A., Kniseley R.N. (1974). Inductively coupled plasmas. Anal. Chem..

[14] Margoshes M., Scribner B.F. (1968). Emission spectrometry. Anal. Chem..

[15] Hammer M.R. (2008). A magnetically excited microwave plasma source for atomic emission spectroscopy with performance approaching that of the inductively coupled plasma. Spectrochim. Acta Part B At. Spectrosc..

[16] Polyakova E., Pelipasov O. (2020). Plasma molecular species and matrix effects in the Hummer cavity microwave induced plasma optical emission spectrometry. Spectrochim. Acta Part B At. Spectrosc..

[17] Xing Z., Wang J., Zhang S., Zhang X. (2009). Determination of bismuth in solid samples by hydride generation atomic fluorescence spectrometry with a dielectric barrier discharge atomizer. Talanta.

[18] Cai Y., Zhang Y.-J., Wu D.-F., Yu Y.-L., Wang J.-H. (2016). Nonthermal optical emission spectrometry: Direct atomization and excitation of cadmium for highly sensitive determination. Anal. Chem..

[19] Anghel S.D., Simon A., Frentiu T. (2005). Characterization of a very low power argon CCP. J. Anal. At. Spectrom..

[20] Sharma T., Litoria P., Bajwa B., Kaur I. (2021). Appraisal of groundwater quality and associated risks in Mansa district (Punjab, India). Environ. Monit. Assess..

[21] Rice G., D’silva A., Fassel V. (1985). A new He discharge-afterglow and its application as a gas chromatographic detector. Spectrochim. Acta Part B At. Spectrosc..

[22] Deng X., Nikiforov A.Y., Vanraes P., Leys C. (2013). Direct current plasma jet at atmospheric pressure operating in nitrogen and air. J. Appl. Phys..

[23] Parzen P., Goldstein L. (1951). Current fluctuations in the direct-current gas discharge plasma. Phys. Rev..

[24] Olsen H. (1963). The electric arc as a light source for quantitative spectroscopy. J. Quant. Spectrosc. Radiat. Transf..

[25] Olsen H. (1959). Thermal and electrical properties of an argon plasma. Phys. Fluids.

[26] Domitz S. (1963). Experimental Evaluation of a Direct-Current Low-Pressure Plasma Source.

[27] Coleman G., Braun W., Allen A. (1980). Characterization of an improved dc plasma excitation source. Appl. Spectrosc..

[28] Tendero C., Tixier C., Tristant P., Desmaison J., Leprince P. (2006). Atmospheric pressure plasmas: A review. Spectrochim. Acta Part B At. Spectrosc..

[29] Pankaj S.K., Wan Z., Keener K.M. (2018). Effects of cold plasma on food quality: A review. Foods.

[30] Reuter S., Von Woedtke T., Weltmann K.-D. (2018). The kINPen—A review on physics and chemistry of the atmospheric pressure plasma jet and its applications. J. Phys. D: Appl. Phys..

[31] Wang X., Shashurin A. (2017). Study of atmospheric pressure plasma jet parameters generated by DC voltage driven cold plasma source. J. Appl. Phys..

[32] Ji H., Dong S., Han F., Li Y., Chen G., Li L., Chen Y. (2018). Effects of dielectric barrier discharge (DBD) cold plasma treatment on physicochemical and functional properties of peanut protein. Food Bioprocess. Technol..

[33] Bourke P., Ziuzina D., Han L., Cullen P., Gilmore B.F. (2017). Microbiological interactions with cold plasma. J. Appl. Microbiol..

[34] Bass A., Chevalier C., Blades M. (2001). A capacitively coupled microplasma (CCµP) formed in a channel in a quartz wafer. J. Anal. At. Spectrom..

[35] Liang D.C., Blades M. (1988). Atmospheric pressure capacitively coupled plasma atomizer for atomic absorption spectrometry. Anal. Chem..

[36] Zou Z., Deng Y., Hu J., Jiang X., Hou X. (2018). Recent trends in atomic fluorescence spectrometry towards miniaturized instrumentation-A review. Anal. Chim. Acta.

[37] Tombrink S., Müller S., Heming R., Michels A., Lampen P., Franzke J. (2010). Liquid analysis dielectric capillary barrier discharge. Anal. Bioanal. Chem..

[38] Hu J., Li W., Zheng C., Hou X. (2011). Dielectric barrier discharge in analytical spectrometry. Appl. Spectrosc. Rev..

[39] Krähling T., Müller S., Meyer C., Stark A.-K., Franzke J. (2011). Liquid electrode dielectric barrier discharge for the analysis of solved metals. J. Anal. At. Spectrom..

[40] Zhu Z., Liu J., Zhang S., Na X., Zhang X. (2008). Determination of Se, Pb, and Sb by atomic fluorescence spectrometry using a new flameless, dielectric barrier discharge atomizer. Spectrochim. Acta Part. B At. Spectrosc..

[41] Niu G., Knodel A., Burhenn S., Brandt S., Franzke J. (2020). Miniature Dielectric Barrier Discharge (DBD) in Analytical Atomic Spectrometry. Anal. Chim. Acta.

[42] Michels A., Tombrink S., Vautz W., Miclea M., Franzke J. (2007). Spectroscopic characterization of a microplasma used as ionization source for ion mobility spectrometry. Spectrochim. Acta Part B At. Spectrosc..

[43] Vautz W., Michels A., Franzke J. (2008). Micro-plasma: A novel ionisation source for ion mobility spectrometry. Anal. Bioanal. Chem..

[44] Brandt S., Klute F.D., Schütz A., Marggraf U., Drees C., Vogel P., Vautz W., Franzke J. (2018). Flexible microtube plasma (FμTP) as an embedded ionization source for a microchip mass spectrometer interface. Anal. Chem..

[45] Jung M.Y., Kang J.H., Choi Y.S., Lee J.Y., Park J.S. (2019). Analytical features of microwave plasma-atomic emission spectrometry (MP-AES) for the quantitation of manganese (Mn) in wild grape (*Vitis coignetiae*) red wines: Comparison with inductively coupled plasma-optical emission spectrometry (ICP-OES). Food Chem..

[46] Sing R., Salin E. (1989). Introduction of liquid samples into the inductively coupled plasma by direct insertion on a wire loop. Anal. Chem..

[47] Buckley B.T., Boss C.B. (1990). A tungsten filament vaporizer for sample introduction into a direct-current plasma. Appl. Spectrosc..

[48] Kleinmann I., Svoboda V. (1969). High-frequency excitation of independently vaporized samples in emission spectrometry. Anal. Chem..

[49] Long S., Snook R., Browner R. (1985). Some observations on electrothermal vaporisation for sample introduction into the inductively coupled plasma. Spectrochim. Acta Part B At. Spectrosc..

[50] Gunn A., Millard D., Kirkbright G. (1978). Optical emission spectrometry with an inductively coupled radiofrequency argon plasma source and sample introduction with a graphite rod electrothermal vaporisation device. Part I. Instrumental assembly and performance characteristics. Analyst.

[51] Qudus H.I., Purwadi P., Holilah I., Hadi S. (2021). Analysis of Mercury in Skin Lightening Cream by Microwave Plasma Atomic Emission Spectroscopy (MP-AES). Molecules.

[52] Okamoto Y. (1994). High-sensitivity microwave-induced plasma mass spectrometry for trace element analysis. J. Anal. At. Spectrom..

[53] Rippetoe W., Johnson E., Vickers T. (1975). Characterization of the plume of a direct current plasma arc for emission spectrometric analysis. Anal. Chem..

[54] Braman R.S., Dynako A. (1968). Direct current discharge spectral emission-type detector. Anal. Chem..

[55] Gard E., Mayer J.E., Morrical B.D., Dienes T., Fergenson D.P., Prather K.A. (1997). Real-time analysis of individual atmospheric aerosol particles: Design and performance of a portable ATOFMS. Anal. Chem..

[56] Chen H., Gamez G., Zenobi R. (2011). What can we learn from ambient ionization techniques?. J. Am. Soc. Mass Spectrom..

[57] Cooks R.G., Ouyang Z., Takats Z., Wiseman J.M. (2006). Ambient mass spectrometry. Science.

[58] Keil A., Talaty N., Janfelt C., Noll R.J., Gao L., Ouyang Z., Cooks R.G. (2007). Ambient mass spectrometry with a handheld mass spectrometer at high pressure. Anal. Chem..

[59] Yadav B., Spinelli A.C., Govindan B.N., Tsui Y.Y., McMullen L.M., Roopesh M. (2019). Cold plasma treatment of ready-to-eat ham: Influence of process conditions and storage on inactivation of Listeria innocua. Food Res. Int..

[60] Rohner U., Whitby J.A., Wurz P. (2003). A miniature laser ablation time-of-flight mass spectrometer for in situ planetary exploration. Meas. Sci. Technol..

[61] Afgan M.S., Hou Z., Wang Z. (2017). Quantitative analysis of common elements in steel using a handheld μ-LIBS instrument. J. Anal. At. Spectrom..

[62] Meuzelaar H.L., McClennen W., Dworzanski J., Sheya S., Snyder A., Harden C., Arnold N. (1995). Hyphenated Techniques: The Next Generation of Field-Portable Analytical Instruments?.

[63] Fantz U. (2006). Basics of plasma spectroscopy. Plasma Sources Sci. Technol..

[64] Chai J., Zhang K., Xue Y., Liu W., Chen T., Lu Y., Zhao G. (2020). Review of MEMS Based Fourier Transform Spectrometers. Micromachines.

[65] Smith J.P. (2000). Product Review: Spectrometers Get Small. Anal. Chem..

[66] Bacon C.P., Mattley Y., DeFrece R. (2004). Miniature spectroscopic instrumentation: Applications to biology and chemistry. Rev. Sci. Instrum..

[67] Cai F., Wang Y., Gao M., He S. (2018). The design and implementation of a low-cost multispectral endoscopy through galvo scanning of a fiber bundle. Opt. Commun..

[68] Sigernes F., Syrjäsuo M., Storvold R., Fortuna J., Grøtte M.E., Johansen T.A. (2018). Do it yourself hyperspectral imager for handheld to airborne operations. Opt. Express.

[69] Crocombe R.A. Handheld spectrometers: The state of the art. Proceedings of the Next-Generation Spectroscopic Technologies VI, 8726, 87260R, International Society for Optics and Photonics.

[70] Frentiu T., Petreus D., Senila M., Mihaltan A.I., Darvasi E., Ponta M., Plaian E., Cordos E.A. (2011). Low power capacitively coupled plasma microtorch for simultaneous multielemental determination by atomic emission using microspectrometers. Microchem. J..

[71] Ye E., Atabaki A.H., Han N., Ram R.J. (2016). Miniature, sub-nanometer resolution Talbot spectrometer. Opt. Lett..

[72] McGonigle A.J., Wilkes T.C., Pering T.D., Willmott J.R., Cook J.M., Mims F.M., Parisi A.V. (2018). Smartphone spectrometers. Sensors.

[73] Jian D., Wang B., Huang H., Meng X., Liu C., Xue L., Liu F., Wang S. (2019). Sunlight based handheld smartphone spectrometer. Biosens. Bioelectron..

[74] You D.J., San Park T., Yoon J.-Y. (2013). Cell-phone-based measurement of TSH using Mie scatter optimized lateral flow assays. Biosens. Bioelectron..

[75] Kim B., Jeon M., Kim Y.-J., Choi S. (2020). Open-source, handheld, wireless spectrometer for rapid biochemical assays. Sens. Actuators B Chem..

[76] Xu W., Manicke N.E., Cooks G.R., Ouyang Z. (2010). Miniaturization of mass spectrometry analysis systems. JALA J. Assoc. Lab. Autom..

[77] Ouyang Z., Cooks R.G. (2009). Miniature mass spectrometers. Annu. Rev. Anal. Chem..

[78] Ouyang Z., Noll R.J., Cooks R.G. (2009). Handheld Miniature Ion Trap Mass Spectrometers. Anal. Chem..

[79] Snyder D.T., Pulliam C.J., Ouyang Z., Cooks R.G. (2016). Miniature and fieldable mass spectrometers: Recent advances. Anal. Chem..

[80] Ren Z., Guo M., Cheng Y., Wang Y., Sun W., Zhang H., Dong M., Li G. (2018). A review of the development and application of space miniature mass spectrometers. Vacuum.

[81] Guo Q., Gao L., Zhai Y., Xu W. (2018). Recent developments of miniature ion trap mass spectrometers. Chin. Chem. Lett..

[82] Miller P.E., Denton M.B. (1986). The quadrupole mass filter: Basic operating concepts. J. Chem. Educ..

[83] Jonscher K.R., Yates III J.R. (1997). The quadrupole ion trap mass spectrometer—A small solution to a big challenge. Anal. Biochem..

[84] Blakeman K. (2015). Development of High Pressure Mass Spectrometry for Handheld Instruments. Ph.D. Dissertation.

[85] Gao L., Song Q., Patterson G.E., Cooks R.G., Ouyang Z. (2006). Handheld rectilinear ion trap mass spectrometer. Anal. Chem..

[86] Turner R.B., Brokenshire J.L. (1994). Hand-held ion mobility spectrometers. TrAC Trends Anal. Chem..

[87] Hoaglund C.S., Valentine S.J., Sporleder C.R., Reilly J.P., Clemmer D.E. (1998). Three-dimensional ion mobility/TOFMS analysis of electrosprayed biomolecules. Anal. Chem..

[88] Hollerbach A., Fedick P.W., Cooks R.G. (2018). Ion mobility–mass spectrometry using a dual-gated 3D printed ion mobility spectrometer. Anal. Chem..

[89] Reinecke T., Clowers B.H. (2018). Implementation of a flexible, open-source platform for ion mobility spectrometry. HardwareX.

[90] Kabir K.M., Donald W.A. (2017). Microscale differential ion mobility spectrometry for field deployable chemical analysis. TrAC Trends Anal. Chem..

[91] Merenbloom S.I., Bohrer B.C., Koeniger S.L., Clemmer D.E. (2007). Assessing the peak capacity of IMS—IMS separations of tryptic peptide ions in He at 300 K. Anal. Chem..

[92] Mielczarek P., Silberring J., Smoluch M. (2020). Miniaturization in mass spectrometry. Mass Spectrom. Rev..

[93] Yang M., Kim T.-Y., Hwang H.-C., Yi S.-K., Kim D.-H. (2008). Development of a palm portable mass spectrometer. J. Am. Soc. Mass Spectrom..

[94] Hendricks P.I., Dalgleish J.K., Shelley J.T., Kirleis M.A., McNicholas M.T., Li L., Chen T.-C., Chen C.-H., Duncan J.S., Boudreau F. (2014). Autonomous in situ analysis and real-time chemical detection using a backpack miniature mass spectrometer: Concept, instrumentation development, and performance. Anal. Chem..

[95] Li L., Chen T.-C., Ren Y., Hendricks P.I., Cooks R.G., Ouyang Z. (2014). Mini 12, Miniature Mass Spectrometer for Clinical and Other Applications—Introduction and Characterization. Anal. Chem..

[96] Maas J.D., Hendricks P.I., Ouyang Z., Cooks R.G., Chappell W.J. (2010). Miniature monolithic rectilinear ion trap arrays by stereolithography on printed circuit board. J. Microelectromech. Syst..

[97] Hendricks P., Duncan J., Noll R.J., Ouyang Z., Cooks R.G. (2011). Performance of a low voltage ion trap. Int. J. Mass Spectrom..

[98] Stravs M.A., Stamm C., Ort C., Singer H. (2021). Transportable Automated HRMS Platform “MS2field” Enables Insights into Water-Quality Dynamics in Real Time. Environ. Sci. Technol. Lett..

[99] Wang J. (2002). Portable electrochemical systems. Trac Trends Anal. Chem..

[100] Motalebizadeh A., Bagheri H., Asiaei S., Fekrat N., Afkhami A. (2018). New portable smartphone-based PDMS microfluidic kit for the simultaneous colorimetric detection of arsenic and mercury. RSC Adv..

[101] Cremers D.A., Ferris M.J., Davies M. (1996). Transportable laser-induced breakdown spectroscopy (LIBS) instrument for field-based soil analysis. Advanced Technologies for Environmental Monitoring and Remediation.

[102] Myers M.J., Myers J.D., Sarracino J.T., Hardy C.R., Guo B., Christian S.M., Myers J.A., Roth F., Myers A.G. (2010). LIBS system with compact fiber spectrometer, head mounted spectra display and hand held eye-safe erbium glass laser gun. Solid State Lasers XIX: Technology and Devices.

[103] Ripoll L., Hidalgo M. (2019). Electrospray deposition followed by laser-induced breakdown spectroscopy (ESD-LIBS): A new method for trace elemental analysis of aqueous samples. J. Anal. At. Spectrom..

[104] Senesi G.S., Harmon R.S., Hark R.R. (2020). Field-portable and handheld LIBS: Historical review, current status and future prospects. Spectrochim. Acta Part B At. Spectrosc..

[105] Borrill A.J., Reily N.E., Macpherson J.V. (2019). Addressing the practicalities of anodic stripping voltammetry for heavy metal detection: A tutorial review. Analyst.

[106] Kruusma J., Banks C.E., Compton R.G. (2004). Mercury-free sono-electroanalytical detection of lead in human blood by use of bismuth-film-modified boron-doped diamond electrodes. Anal. Bioanal. Chem..

[107] Kalnicky D.J., Singhvi R. (2001). Field portable XRF analysis of environmental samples. J. Hazard. Mater..

[108] Zhou S., Yuan Z., Cheng Q., Zhang Z., Yang J. (2018). Rapid in situ determination of heavy metal concentrations in polluted water via portable XRF: Using Cu and Pb as example. Environ. Pollut..

[109] You R., Li P., Jing G., Cui T. (2019). Ultrasensitive micro ion selective sensor arrays for multiplex heavy metal ions detection. Microsyst. Technol..

[110] Szigeti Z., Vigassy T., Bakker E., Pretsch E. (2006). Approaches to improving the lower detection limit of polymeric membrane ion-selective electrodes. Electroanal. Int. J. Devoted Fundam. Pract. Asp. Electroanal..

[111] Sobin C., Parisi N., Schaub T., de la Riva E. (2011). A Bland–Altman comparison of the Lead Care^®^ System and inductively coupled plasma mass spectrometry for detecting low-level lead in child whole blood samples. J. Med Toxicol..

[112] Mason J., Ortiz D., Pappas S., Quigley S., Yendell S., Ettinger A.S. (2019). Response to the US FDA LeadCare Testing Systems Recall and CDC Health Alert. J. Public Health Manag. Pract. JPHMP.

[113] Montaño M.D., Olesik J.W., Barber A.G., Challis K., Ranville J.F. (2016). Single Particle ICP-MS: Advances toward routine analysis of nanomaterials. Anal. Bioanal. Chem..

[114] Mueller L., Traub H., Jakubowski N., Drescher D., Baranov V.I., Kneipp J. (2014). Trends in single-cell analysis by use of ICP-MS. Anal. Bioanal. Chem..

[115] Barreiros M., Carvalho M., Costa M., Marques M., Ramos M. (1997). Application of total reflection XRF to elemental studies of drinking water. X-ray Spectrom. Int. J..

[116] Kang J., Li R., Wang Y., Chen Y., Yang Y. (2017). Ultrasensitive detection of trace amounts of lead in water by LIBS-LIF using a wood-slice substrate as a water absorber. J. Anal. At. Spectrom..

[117] Wang J. (2005). Stripping analysis at bismuth electrodes: A review. Electroanal. Int. J. Devoted Fundam. Pract. Asp. Electroanal..

[118] Xing G., Sardar M.R., Lin B., Lin J.-M. (2019). Analysis of trace metals in water samples using NOBIAS chelate resins by HPLC and ICP-MS. Talanta.

[119] Milne A., Landing W., Bizimis M., Morton P. (2010). Determination of Mn, Fe, Co, Ni, Cu, Zn, Cd and Pb in seawater using high resolution magnetic sector inductively coupled mass spectrometry (HR-ICP-MS). Anal. Chim. Acta.

[120] Ma X., Zhang Z. (2004). Wavelet smoothing applied to the determination of trace arsenic, lead, antimony and selenium in environmental water by ICP-OES. J. Anal. At. Spectrom..

[121] Bakker E., Pretsch E. (2007). Modern potentiometry. Angew. Chem. Int. Ed..

[122] Bu L., Xie Q., Ming H. (2020). Simultaneous sensitive analysis of Cd (ii), Pb (ii) and As (iii) using a dual-channel anodic stripping voltammetry approach. New J. Chem..

[123] Tanvir E., Whitfield K.M., Ng J.C., Shaw P.N. (2020). Development and validation of an ICP-MS method and its application to determine multiple trace elements in small volumes of whole blood and plasma. J. Anal. Toxicol..

[124] Massadeh A., Gharibeh A., Omari K., Al-Momani I., Alomari A., Tumah H., Hayajneh W. (2010). Simultaneous determination of Cd, Pb, Cu, Zn, and Se in human blood of Jordanian smokers by ICP-OES. Biol. Trace Elem. Res..

[125] Soleymanpour A., Shafaatian B., Kor K., Hasaninejad A.R. (2012). Coated wire lead (II)-selective electrode based on a Schiff base ionophore for low concentration measurements. Mon. Für Chem. Chem. Mon..

[126] Landes F.C., Paltseva A., Sobolewski J.M., Cheng Z., Ellis T.K., Mailloux B.J., van Geen A. (2019). A field procedure to screen soil for hazardous lead. Anal. Chem..

[127] Li X., Coles B.J., Ramsey M.H., Thornton I. (1995). Sequential extraction of soils for multielement analysis by ICP-AES. Chem. Geol..

[128] Rehan I., Gondal M., Rehan K. (2018). Determination of lead content in drilling fueled soil using laser induced spectral analysis and its cross validation using ICP/OES method. Talanta.

[129] Kadachi A.N., Al-Eshaikh M.A. (2012). Limits of detection in XRF spectroscopy. X-ray Spectrom..

[130] Tian K., Huang B., Xing Z., Hu W. (2018). In situ investigation of heavy metals at trace concentrations in greenhouse soils via portable X-ray fluorescence spectroscopy. Environ. Sci. Pollut. Res..

[131] Kadara R.O., Tothill I.E. (2004). Stripping chronopotentiometric measurements of lead (II) and cadmium (II) in soils extracts and wastewaters using a bismuth film screen-printed electrode assembly. Anal. Bioanal. Chem..

[132] Riondato J., Vanhaecke F., Moens L., Dams R. (2000). Fast and reliable determination of (ultra-) trace and/or spectrally interfered elements in water by sector field ICP-MS. J. Anal. At. Spectrom..

[133] Fichet P., Tabarant M., Salle B., Gautier C. (2006). Comparisons between libs and ICP/OES. Anal. Bioanal. Chem..

[134] Mages M., Woelfl S., Óvári M. (2003). The use of a portable total reflection X-ray fluorescence spectrometer for field investigation. Spectrochim. Acta Part B At. Spectrosc..

[135] Kallithrakas-Kontos N., Koulouridakis P., Hatzistavros V., Aretaki I. (2009). Chromium speciation by TXRF analysis. X-ray Spectrom. Int. J..

[136] Leśniewska B., Godlewska-Żyłkiewicz B. (2019). Speciation of chromium in alkaline soil extracts by an ion-pair reversed phase HPLC-ICP MS method. Molecules.

[137] Mamatha P., Venkateswarlu G., Swamy A., Sahayam A. (2014). Microwave assisted extraction of Cr (III) and Cr (VI) from soil/sediments combined with ion exchange separation and inductively coupled plasma optical emission spectrometry detection. Anal. Methods.

[138] Dell’Aglio M., Gaudiuso R., Senesi G.S., De Giacomo A., Zaccone C., Miano T.M., De Pascale O. (2011). Monitoring of Cr, Cu, Pb, V and Zn in polluted soils by laser induced breakdown spectroscopy (LIBS). J. Environ. Monit..

[139] Hartyani Z., Dávid E., Szabó S., Szilágyi V., Horváth T., Tóth Á.H. (2000). Determination of the trace elements distribution of polluted soils in Hungary by X-ray methods. Microchem. J..

[140] Terán-Baamonde J., Soto-Ferreiro R.-M., Carlosena A., Andrade J.-M., Prada D. (2018). Determination of cadmium in sediments by diluted HCI extraction and isotope dilution ICP-MS. Talanta.

[141] Floyd M., Fassel V., D’silva A. (1980). Computer-controlled scanning monochromator for the determination of 50 elements in geochemical and environmental samples by inductively coupled plasma-atomic emission spectrometry. Anal. Chem..

[142] Santos D., Nunes L.C., Trevizan L.C., Godoi Q., Leme F.O., Braga J.W.B., Krug F.J. (2009). Evaluation of laser induced breakdown spectroscopy for cadmium determination in soils. Spectrochim. Acta Part B At. Spectrosc..

[143] Knopp R., Scherbaum F., Kim J. (1996). Laser induced breakdown spectroscopy (LIBS) as an analytical tool for the detection of metal ions in aqueous solutions. Fresenius’ J. Anal. Chem..

[144] Marguí E., Queralt I., Hidalgo M. (2013). Determination of cadmium at ultratrace levels in environmental water samples by means of total reflection X-ray spectrometry after dispersive liquid–liquid microextraction. J. Anal. At. Spectrom..

[145] Essi M., Kouame N., Cisse G. (2018). Cd-ISe and membrane technique device for in situ monitoring. Chalcogenide Lett..

[146] Venus M., Puntarić D., Gvozdić V., Vidosavljević D., Bijelić L., Puntarić A., Puntarić E., Vidosavljević M., Matijana J., Jasenka Š. (2019). Determinations of uranium concentrations in soil, water, vegetables and biological samples from inhabitants of war affected areas in eastern Croatia (ICP-MS method). J. Environ. Radioact..

[147] Daneshvar G., Jabbari A., Yamini Y., Paki D. (2009). Determination of uranium and thorium in natural waters by ICP-OES after on-line solid phase extraction and preconcentration in the presence of 2, 3-dihydro-9, 10-dihydroxy-1, 4-antracenedion. J. Anal. Chem..

[148] Sarkar A., Alamelu D., Aggarwal S.K. (2008). Determination of thorium and uranium in solution by laser-induced breakdown spectrometry. Appl. Opt..

[149] Alsecz A., Osan J., Kurunczi S., Alföldy B., Varhegyi A., Török S. (2007). Analytical performance of different X-ray spectroscopic techniques for the environmental monitoring of the recultivated uranium mine site. Spectrochim. Acta Part B At. Spectrosc..

[150] Agrahari S., Kumar S., Srivastava A. (2014). Ion selective electrode for uranium based on composite multiwalled carbon nanotube-benzo-15-crown-5 in PVC matrix coated on graphite rod. J. Anal. Chem..

[151] Trivelpiece A., Gould R. (1959). Space charge waves in cylindrical plasma columns. J. Appl. Phys..

[152] Moisan M., Beaudry C., Lepprince P. (1974). A new HF device for the production of long plasma columns at a high electron density. Phys. Lett. A.

[153] Moisan M., Leprince P., Beaudry C., Bloyet E. (1977). Devices and Methods of Using HF Waves to Energize a Column of Gas Enclosed in an Insulating Casing. U.S. Patent.

[154] Moisan M., Beaudry C., Leprince P. (1975). A small microwave plasma source for long column production without magnetic field. IEEE Trans. Plasma Sci..

[155] Moisan M., Zakrzewski Z., Pantel R. (1979). The theory and characteristics of an efficient surface wave launcher (surfatron) producing long plasma columns. J. Phys. D Appl. Phys..

[156] Selby M., Hieftje G.M. (1987). Taming the surfatron. Spectrochim. Acta Part B At. Spectrosc..

[157] Long G.L., Perkins L.D. (1987). Direct introduction of aqueous samples into a low-powered microwave-induced plasma for atomic emission spectrometry. Appl. Spectrosc..

[158] Deng Y., Zeng W., Jiang X., Hou X. (2020). Portable photochemical vapor generation-microwave plasma optical emission spectrometer. J. Anal. At. Spectrom..

[159] Haertel B., Von Woedtke T., Weltmann K.-D., Lindequist U. (2014). Non-thermal atmospheric-pressure plasma possible application in wound healing. Biomol. Ther..

[160] Keidar M. (2015). Plasma for cancer treatment. Plasma Sources Sci. Technol..

[161] Misra N., Moiseev T., Patil S., Pankaj S., Bourke P., Mosnier J., Keener K., Cullen P. (2014). Cold plasma in modified atmospheres for post-harvest treatment of strawberries. Food Bioprocess Technol..

[162] Pavlovich M.J., Clark D.S., Graves D.B. (2014). Quantification of air plasma chemistry for surface disinfection. Plasma Sources Sci. Technol..

[163] Vasilyak L. (2021). Physical methods of disinfection (a review). Plasma Phys. Rep..

[164] Moon S.Y., Choe W. (2003). A comparative study of rotational temperatures using diatomic OH, O_2_ and N^2+^ molecular spectra emitted from atmospheric plasmas. Spectrochim. Acta Part B At. Spectrosc..

[165] Workman J.M., Fleitz P., Fannin H.B., Caruso J.A., Seliskar C. (1988). A comparative study of rotational temperatures in a microwave plasma: OH radical versus N^2+^. Appl. Spectrosc..

[166] Sysolyatina E., Vasiliev M., Kurnaeva M., Kornienko I., Petrov O., Fortov V., Gintsburg A., Petersen E., Ermolaeva S. (2016). Frequency of cell treatment with cold microwave argon plasma is important for the final outcome. J. Phys. D Appl. Phys..

[167] Duarte S., Kuo S., Murata R., Chen C., Saxena D., Huang K., Popovic S. (2011). Air plasma effect on dental disinfection. Phys. Plasmas.

[168] Iuchi K., Morisada Y., Yoshino Y., Himuro T., Saito Y., Murakami T., Hisatomi H. (2018). Cold atmospheric-pressure nitrogen plasma induces the production of reactive nitrogen species and cell death by increasing intracellular calcium in HEK293T cells. Arch. Biochem. Biophys..

[169] Motley C.B., Long G.L. (1990). Evaluation of sample introduction techniques of packed-column SFC into an MIP. Appl. Spectrosc..

[170] Wind M., Eisenmenger A., Lehmann W.D. (2002). Modified direct injection high efficiency nebulizer with minimized dead volume for the analysis of biological samples by micro-and nano-LC-ICP-MS. J. Anal. At. Spectrom..

[171] Takats Z., Wiseman J.M., Cooks R.G. (2005). Ambient mass spectrometry using desorption electrospray ionization (DESI): Instrumentation, mechanisms and applications in forensics, chemistry, and biology. J. Mass Spectrom..

[172] Jiang S., Houk R. (1986). Arc nebulization for elemental analysis of conducting solids by inductively coupled plasma mass spectrometry. Anal. Chem..

[173] Brenner I., Lorber A., Goldbart Z. (1987). Trace element analysis of geological materials by direct solids insertion of a graphite cup into an inductively coupled plasma. Spectrochim. Acta Part B At. Spectrosc..

